# Design of non-equiatomic medium-entropy alloys

**DOI:** 10.1038/s41598-018-19449-0

**Published:** 2018-01-19

**Authors:** Yang Zhou, Dong Zhou, Xi Jin, Lu Zhang, Xingyu Du, Bangsheng Li

**Affiliations:** 1 School of Materials Science and Engineering, Harbin Institute of Technology, Harbin, 150001 China; 2 State Key Laboratory of Advanced Welding and Joining, Harbin Institute of Technology, Harbin, 150001 China; 3 Micro/Nano Technology Research Center, Harbin Institute of Technology, Harbin, 150001 China

## Abstract

High-entropy alloys have attracted much attention due to their unique microstructures and excellent properties. Since their invention more than ten years ago, research attention has been mainly focused on the study of multicomponent alloys with equiatomic or near-equiatomic compositions. Here we propose a novel design of non-equiatomic medium-entropy alloys that contain one matrix element and several equiatomic alloying elements. To verify the utility of this new design, a series of Co-free Fe_x_(CrNiAl)_100−x_ (at.%, 25 ≤ x ≤ 65) medium-entropy alloys were designed from the much-studied FeNiCrCoAl high-entropy alloy. Detailed characterization reveals that the alloys exhibit novel two-phase microstructures consisting of B2-ordered nanoprecipitates and BCC-disordered matrix. As the alloys deviate far from equiatomic composition, the structure of the nanoprecipitates transfers from a spinodal-like intertwined structure to a nanoparticle dispersed structure. Previous parametric approaches to predict phase formation rules for high-entropy alloys are unable to describe the phase separation behaviors in the studied alloys. Our findings provide a new route to design medium-entropy alloys and also demonstrate a strategy for designing nanostructured alloys from multicomponent alloy systems through simple variations in non-equiatomic compositions.

## Introduction

High-entropy alloys (HEAs) are a newly emerging class of multicomponent alloys that have attracted widespread attention over the past decade^1–6^. Such alloys were initially defined as those containing five or more principal elements in equimolar or near-equimolar ratios, each element with a concentration being between 5 at.% and 35 at.%^1^. Later, another definition of HEAs was also proposed, which suggests that the alloys can be classified as HEAs if their configurational entropies are higher than 1.5 *R* (*R* is the gas constant)^3,4^. It can be known from the two definitions that the basic principle behind the design of HEAs is to have high configurational entropy so as to stabilize solid solutions rather than intermetallic compounds. Owing to the high entropy effect, a lot of HEAs can form simple body-centered cubic (BCC) and/or face-centered cubic (FCC) solid solution microstructures^7–12^. Consequently, many HEAs have been found to exhibit excellent properties such as high strength, good wear resistance, outstanding corrosion resistance, and exceptional high and low temperature performance, having a variety of promising applications^13–19^.

However, not all HEAs can form a single phase solid solution microstructure. In many cases, the formation of ordered intermetallic phases, complicated compounds or even amorphous phases was often observed^20–23^.The formation rules of different phases in HEAs have been widely studied for many years, but the factors controlling phase formation are still under debate^24–29^. In order to optimize the microstructure for desired properties, a common strategy employed until now has been to design multicomponent alloys with equiatomic or near-equiatomic compositions. Recently, some attention has been shifted to the study of multicomponent alloys with non-equiatomic compositions^30–40^. For example, some non-equiatomic multicomponent alloys have been reported, such as Fe_42_Mn_20_Ni_30_Co_6_Cr_2_ and Fe_40_Mn_27_Ni_26_Co_5_Cr_2_ quinary alloys^37,38^, Cr_10_Mn_40_Fe_40_Co_10_ and Cr_4_Mn_28_Fe_40_Ni_28_ quaternary alloys^39,40^. The configurational entropies of these alloys at a random state are between 1 *R* and 1.5 *R*, which means that they should be classified as medium-entropy alloys (MEAs)^3,4^. Despite of the reduced configurational entropies, these MEAs possess excellent microstructures and properties, which can be compered with or even better than those of HEAs. Not only did this urge us to focus our attention on the class of MEAs, it also triggered us to develop a general approach for designing non-equiatomic MEAs. In the present work, we proposed a novel design of non-equiatomic MEAs that contain one matrix element and several equiatomic alloying elements. In this paper, we firstly expounded the idea and basis of this new alloy design. Then, to verify the utility of this design, we designed a series of Co-free Fe_*x*_(CrNiAl)_100−*x*_ (at. %, *x* = 25, 35, 45, 55 and 65) MEAs from the much-studied FeNiCrCoAl HEA. After that, we systematically studied the microstructures of these alloys and also discussed about their phase separation behaviors.

## Results

We start expounding our design idea from the classification of the alloy world based on the ranges of configurational entropy (Δ*S*_*mix*_). The alloy world can be divided into three fields, including low-entropy alloys (LEAs, Δ*S*_*mix*_ < 1 *R*), medium-entropy alloys (MEAs, 1 *R* < Δ*S*_*mix*_ < 1.5 *R*) and high-entropy alloys (HEAs, Δ*S*_*mix*_ > 1.5 *R*)^3,4^. For the classification, the values of Δ*S*_*mix*_ are calculated at a random state, no matter the alloys are single phase or multiphase at room temperature^3,4^. According to Boltzmann’s hypothesis, for a *n*-element multicomponent alloy at a random state the Δ*S*_*mix*_ can be calculated by the following equation:

1 $${\rm{\Delta }}{S}_{mix}=-R\sum _{i=1}^{n}({x}_{i}\,\mathrm{ln}\,{x}_{i})$$

where the gas constant *R* has a value of 8.314 JK^−1^ mol^−1^, *n* is the number of total elements, *x*_*i*_ is the concentration of element *i*.

When there is one element with atomic percentage of *x* and the other elements have equal atomic percentage, equation (1) will be as following:

2 $${\rm{\Delta }}{S}_{mix}=-R[x\mathrm{ln}\,x+(1-x)\mathrm{ln}(\frac{1-x}{n-1})]$$

**Figure 1:**
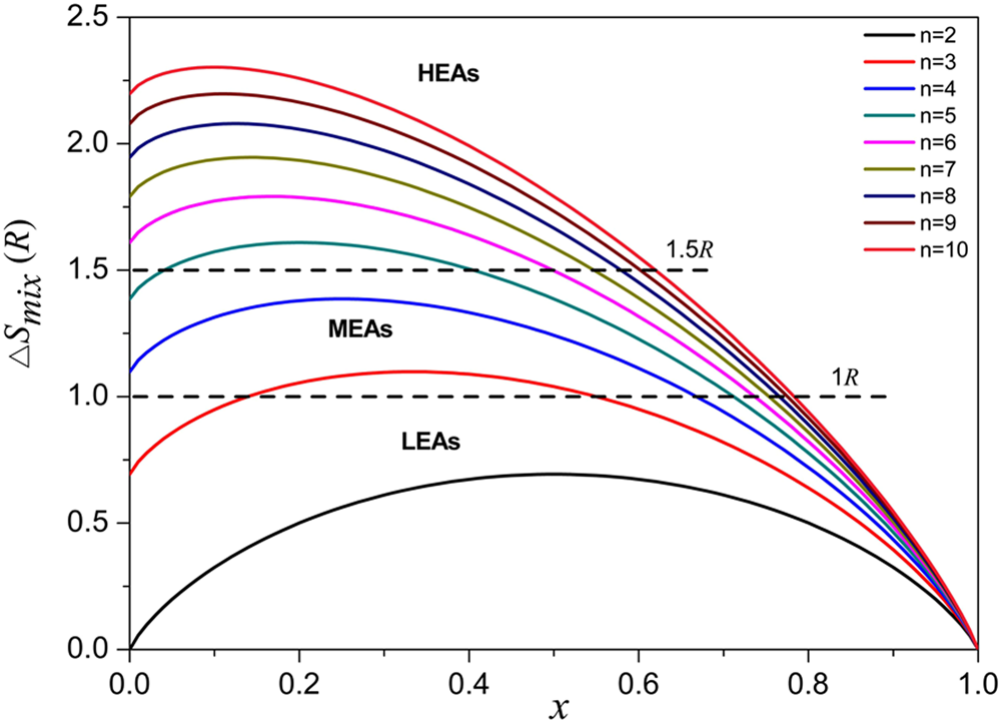
Relationship between Δ*S*_*mix*_ and *x* of *n*-element multicomponent alloys with one matrix element (*n* = 2~10).

Based on equation (2), the relationship between Δ*S*_*mix*_ and *x* of *n*-element multicomponent alloys can be plotted as shown in Fig. 1. It can be seen that many non-equiatomic multicomponent alloys can be regarded as MEAs since their configurational entropies are between *R* and 1.5 *R*. When the multicomponent alloys has one matrix element (*x* > 0.5), only a little proportion of them are HEAs but quite a few are MEAs. Thus, we can design MEAs not only from equiatomic ternary or quaternary alloy systems, but also from non-equiatomic multicomponent alloys in which there can be one matrix element. For example, we can easily design Fe-based Fe_55_Mn_15_Ni_15_Co_15_ (1.18 *R*) quaternary MEA, Fe-based Fe_60_Mn_10_Ni_10_Cr_10_Co_10_ (1.23 *R*) quinary MEA, and Ni-based Ni_60_Fe_8_Cr_8_Co_8_Cu_8_Al_8_ MEA (1.32 *R*) senary MEA from the existing MEA and HEA systems.

To develop a general approach, we advise a new design of non-equiatomic MEAs that contain one matrix element and several equiatomic alloying elements, while also having configurational entropies between 1 *R* and 1.5 *R* at a random state. Based on this design, the range of matrix composition can be up to 66 at.%, 71 at.% and 73 at.% for quaternary, quinary and senary systems, respectively. The concentrations of alloying elements are proposed to have equiatomic ratios, such that they may contribute the most to configurational entropies; however, for a more generalised design it is not a must, as long as the alloys still have medium entropies. In this sense, many non-equiatomic multicomponent alloys such as Fe-based Fe_55_Mn_20_Cr_15_Ni_5_Al_5_ (1.23 *R*) and Ni-based Ni_57_Cr_20_Co_10_Al_8_Mo_3_Ti_2_ (1.26 *R*) can also be regarded as non-equiatomic MEAs. Some conventional alloys containing high concentrations of alloying elements can also be called as non-equiatomic MEAs, such as 316 steel (1.15 *R*), Inconel 718 superalloy (1.31 *R*), Stellite 6 superalloy (1.13 *R*) and Zr_53_Ti_5_Cu_16_Ni_10_Al_16_ amorphous alloy (1.30 *R*)^3,4^.

In the design of non-equiatomic MEAs, we may obtain different phases depending on different alloy compositions. This is supposed to be similar with that in HEA systems. Recently, it has been found that the phase formation rules of HEAs are not only determined by configurational entropy, but they are also controlled by many other factors such as mismatch entropy, mixing enthalpy, atomic size difference, valence and electronegativity^24–29^. According to the previous literatures, solid solution phases are stable only when the HEAs have near-zero values of mixing enthalpy and small atomic size difference^25^. If the mixing enthalpy is negative enough to overcome the entropy effect, the formation of intermetallic compounds will occur^27^. With a more negative mixing enthalpy and a larger atomic size difference, the HEAs may be driven to form amorphous phases^26,29^. It is anticipated, therefore, that a proper design of compositions is also required to achieve desired microstructures and properties for non-equiatomic MEAs.

To verify the utility of this new design, we take the design of Fe-Cr-Ni-Al alloys as an example. This alloy system is chosen from the much-studied Fe-Cr-Co-Ni-Al HEA system^8,12,16,22^. The equiatomic FeCrCoNiAl HEA exhibits a two-phase microstructure consisting of BCC-disordered Fe-Cr-rich precipitates and B2-ordered Ni-Al-rich matrix, while the Co element is uniformly distributed in this alloy^22^. In our alloy design, the expensive Co element is omitted considering that the absence of this element may have little influence on the microstructure due to its uniform distribution. Since the Ni-Al-rich intermetallic phase is hard but brittle, a reverse microstructure that contains Ni-Al-rich precipitates dispersed in Fe-Cr-rich matrix should be better for mechanical properties^8^. In order to achieve this, we set Fe as the matrix element and keep the other alloying elements in equimolar ratios. As a result, a series of Co-free Fe_*x*_(CrNiAl)_100−*x*_ (at. %, *x* = 25, 35, 45, 55 and 65) alloys were designed, which are denoted as Fe25, Fe35, Fe45, Fe55 and Fe65, respectively. The configurational entropies of these alloys at random state are 1.39 *R*, 1.36 *R*, 1.29 *R*, 1.18 *R* and 1.03 *R*, respectively. Therefore, the five alloys can be classified as MEAs since their configurational entropies are between 1 *R* and 1.5 *R*. Specifically, the Fe55 and Fe66 MEAs have a matrix element with atomic percentage higher than 50%. In the following, we will systematically study the microstructures of these MEAs as well as their phase separation behaviors.

**Figure 2:**
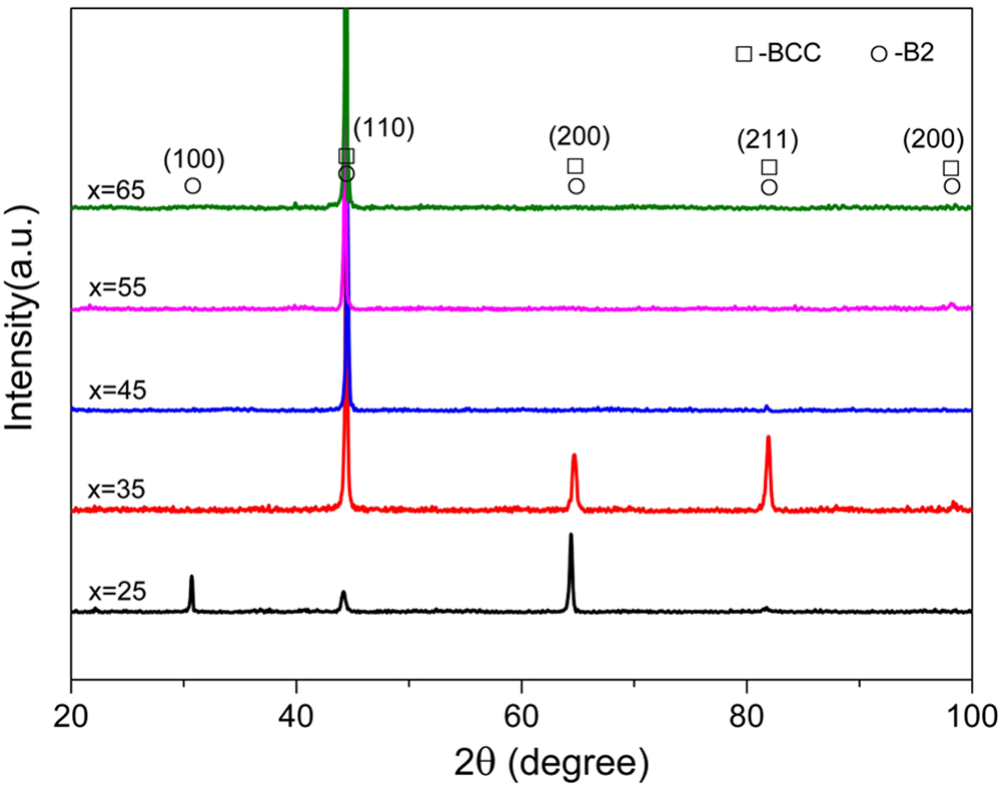
XRD patterns of the Fe_x_(CrNiAl)_100−x_ alloys.

Figure 2 shows the X-ray diffractometer (XRD) patterns of the as-cast Fe_*x*_(CrNiAl)_100−*x*_ alloys. In the Fe25 alloy, two different BCC structures were detected. One is disordered BCC structure and the other is ordered BCC (designated as B2) structure. The appearance of (100) superlattice diffraction peak is attributed to the ordered composition distribution in the B2 structure. The other diffraction peaks of BCC and B2 structures overlap, which indicates that they have similar lattice parameters. For the Fe35 alloy, the diffraction intensity of peaks significantly reduces, but several peaks corresponding to BCC structure can still be observed. In the other three alloys including Fe45, Fe55 and Fe65, only (100) diffraction peaks were detected. Such unusual diffraction phenomena is probably attributed to the (100) preferred orientation in their microstructures. From microstructural characterization below, it can be found that these three alloys also contain two different phases. Based on the results of XRD, it can be concluded that although the Fe_*x*_(CrNiAl)_100−*x*_ alloys have lower and lower mixing entropies when their compositions deviate far from equiatomic compositions, their crystal structures remain quite simple and no complicated compounds appear in these alloys. Hence, the limitation of equiatomic or near-equiatomic compositions in the design of MEAs is not necessary.

**Figure 3:**
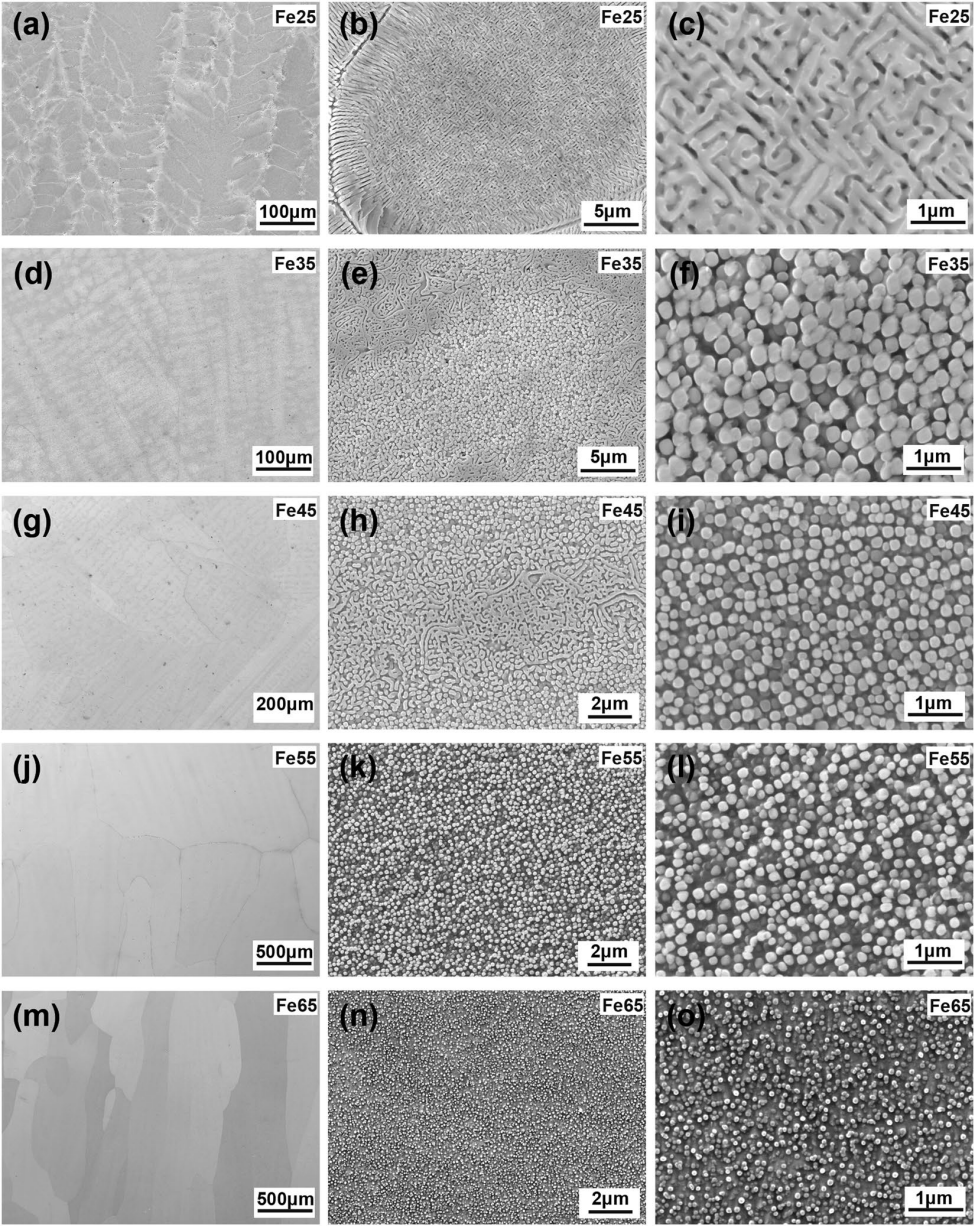
SEM secondary electron images of the Fe_*x*_(CrNiAl)_100−*x*_ alloys. (**a**)~(**c**) *x* = 25, (**d**)~(**f**) *x* = 35, (**g**)~(**i**) *x* = 45, (**j**)~(**l**) *x* = 55, (**m**)~(**o**) *x* = 65.

The microstructures of the Fe_*x*_(CrNiAl)_100−*x*_ alloys were characterized by using scanning electron microscopy (SEM). As is seen from Fig. 3a, the Fe25 alloy exhibits a dendritic microstructure at low magnification. At the edge of dendrites, eutectic lamellas can be observed (Fig. 3b). At high magnification, a spinodal-like intertwined nanostructure is found inside the dendrites (Fig. 3c). In the Fe35 alloy, a typical dendritic microstructure can be also observed (Fig. 3d). The interdendrites exhibit a intertwined nanostructure which seems to form by coalescence of nanoparticles and nanowires (Fig. 3e), while the dendrites contain a large number of nanoparticles dispersed in the matrix (Fig. 3f). The average size of the nanoparticles in this alloy is about 400 nm. Compared with the Fe35 alloy, the Fe45 alloy has a similar dendritic microstructure (Fig. 3g), but the proportion of interdendrites in this alloy becomes smaller (Fig. 3h). In the dendrites of the Fe45 alloy, a large number of well-dispersed nanoparticles can also be observed (Fig. 3i), which are about 250 nm in size. In the Fe55 alloy, a equiaxed microstructure containing large grains up to several millimeters in size can be found (Fig. 3j). There are a large number of uniform nanoparticles dispersed inside the grains (Fig. 3k and l). The average size of the nanoparticles in this alloy are about 200 nm. The microstructure of the Fe65 alloy is similar as that of the Fe55 alloy. Large grains about several millimeters in length can be found in this alloy (Fig. 3m). There are also a large number of well-dispersed nanoparticles inside the grains, which are about 100 nm in size (Fig. 3n and o).

**Figure 4:**
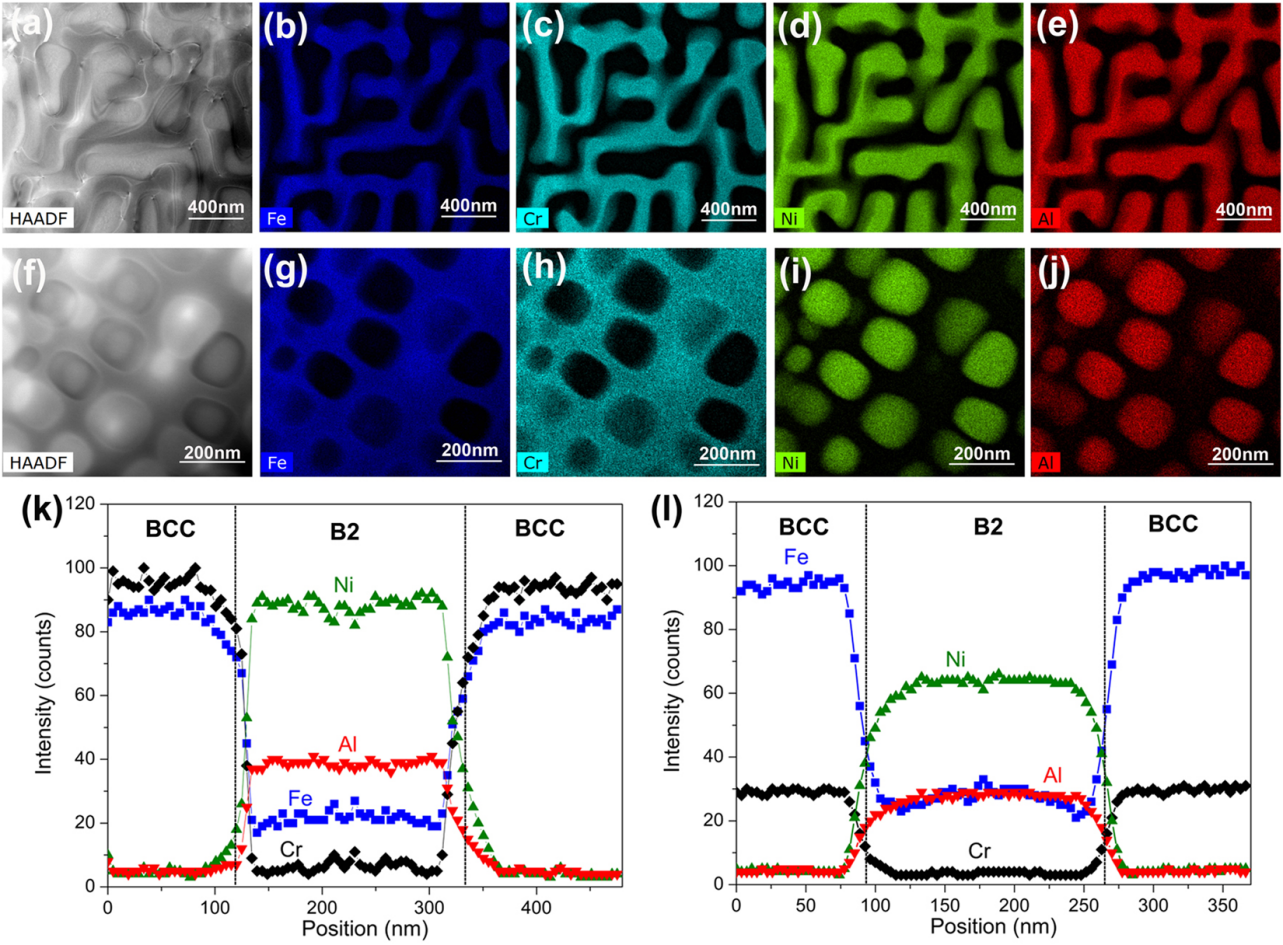
HAADF images, STEM-EDS maps and compositional profiles of the Fe_25_(CrNiAl)_75_ alloy and Fe_55_(CrNiAl)_45_ alloy. (**a**) HAADF image of the Fe_25_(CrNiAl)_75_ alloy, (**b**)~(**e**) STEM-EDS maps of the Fe_25_(CrNiAl)_75_ alloy, (**f**) HAADF image of the Fe_55_(CrNiAl)_45_ alloy, (**g**)~(**j**) STEM-EDS maps of the Fe_55_(CrNiAl)_45_ alloy, (**k**) Compositional profiles across the B2 phase of in the Fe_25_(CrNiAl)_75_, (**l**) Compositional profiles across the B2 phase of in the Fe_55_(CrNiAl)_45_ alloy.

In order to reveal the chemical compositions of the precipitates and the matrix in the Fe_*x*_(CrNiAl)_100−*x*_ alloys, the Fe25 and Fe55 alloys were chosen to be analyzed by high-angle annular dark-field (HAADF) detector and energy dispersive spectrometry (EDS) in a transmission electron microscopy (TEM). Fig. 4 presents the HAADF images, EDS maps and EDS line profiles taken in the two alloys. From images as shown in Fig. 4a, it can be seen that the Fe25 alloy exhibits a intertwined nanostructure, in which the Ni-Al-rich phase and the Fe-Cr-rich phase alternate with a period of about 200 nm in the width. In the Fe55 alloy, the nanoparticles are Ni-Al-rich phase while the matrix is the Fe-Cr-rich phase (Fig. 4b). From the EDS line profiles as shown in Fig. 4c, it can be found that quite a few concentrations of Fe are dissolved in the Ni-Al-rich phase, while only a few of Ni and Al elements are dissolved in the Fe-Cr-rich phase. Compared with the Fe25 alloy, there is more concentration of Fe element dissolved in the Ni-Al-rich phase of the Fe55 alloy. The detailed chemical compositions of the Ni-Al-rich phase and the Fe-Cr-rich phase in the two alloys are listed in Table 1.

**Table 1:** Chemical compositions of phases in Fe_25_(CrNiAl)_75_ and Fe_55_(CrNiAl)_45_ alloys.

alloy	phase	Fe (at.%)	Cr (at.%)	Ni (at.%)	Al (at.%)
Fe_25_(CrNiAl)_75_	Fe-Cr-rich phase	43.31 ± 3.98	49.86 ± 4.57	2.43 ± 0.29	4.40 ± 0.55
	Ni-Al-rich phase	11.03 ± 1.07	2.61 ± 0.31	51.11 ± 4.67	35.25 ± 3.32
Fe_55_(CrNiAl)_45_	Fe-Cr-rich phase	70.30 ± 6.42	21.60 ± 1.95	3.70 ± 0.31	4.40 ± 0.57
	Ni-Al-rich phase	19.43 ± 1.83	2.04 ± 0.27	50.40 ± 4.62	28.13 ± 2.70

**Figure 5:**
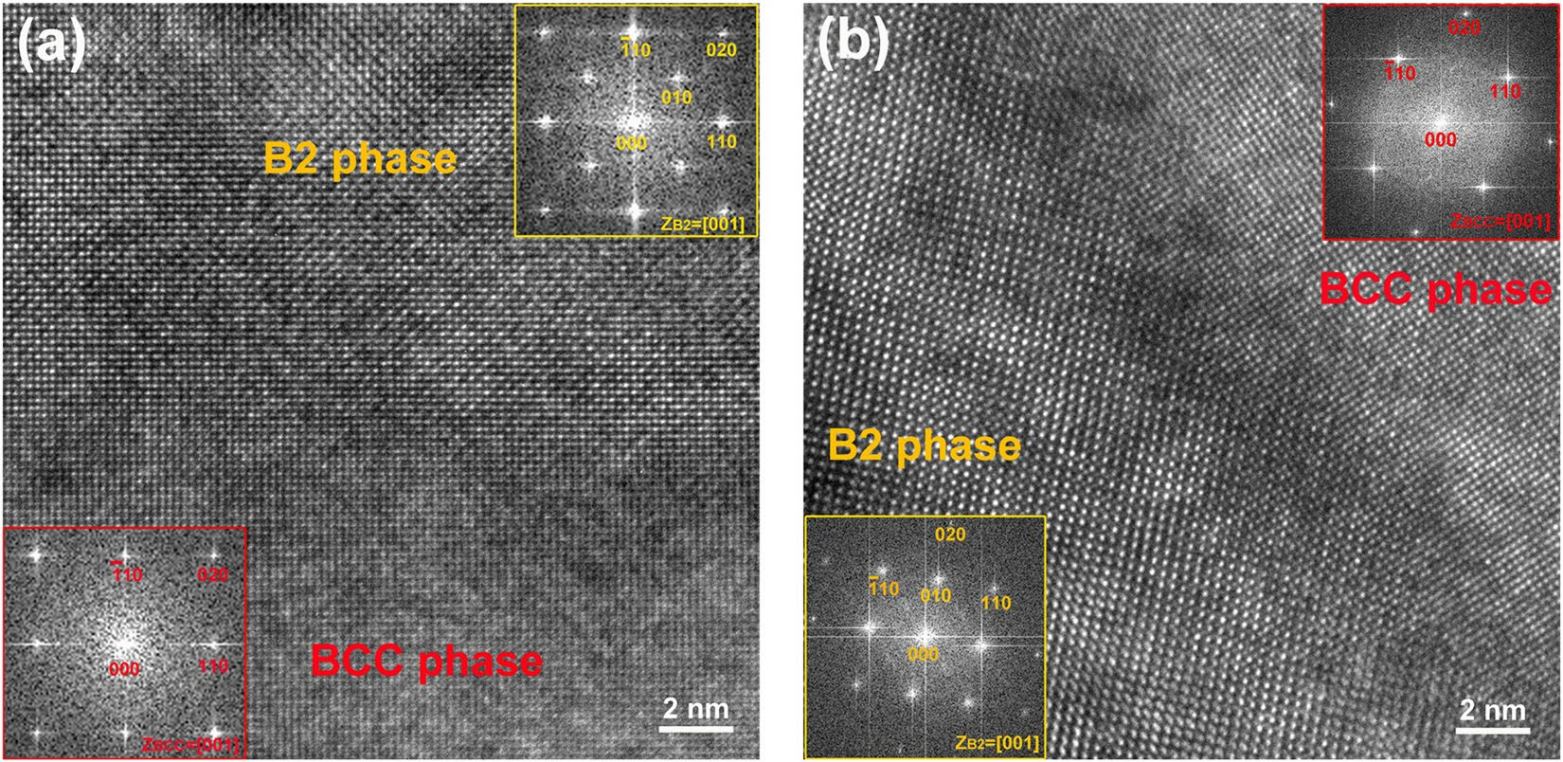
HRTEM images and FFT patterns of B2 and BCC phases in the Fe_25_(CrNiAl)_75_ alloy and Fe_55_(CrNiAl)_45_ alloy. (**a**) HRTEM image of the Fe_25_(CrNiAl)_75_ alloy and the corresponding FFT patterns of B2 and BCC phases in the insets, (**b**) HRTEM image of the Fe_55_(CrNiAl)_45_ alloy and the corresponding FFT patterns of B2 and BCC phases in the insets.

The structures of the Ni-Al-rich and Fe-Cr-rich phases in the Fe25 and Fe55 alloys were further determined by high-resolution transmission electron microscopy (HRTEM) analysis. Fig. 5a shows the HRTEM image and corresponding inverse Fourier transform (IFF) patterns of the phases in the Fe25 alloy. It can be confirmed that the Ni-Al-rich phase is a B2-ordered phase while the Fe-Cr-rich phase is a BCC-disordered phase. The two phases have coherent lattices. These features are consistent with the result of XRD analysis as shown in Fig. 2. As is seen from Fig. 5b, the Fe55 alloy also has two coherent phases, including Ni-Al-rich B2-ordered phase and Fe-Cr-rich BCC-disordered phase. However, the degree of order of the B2 phases in the alloys is still unable to be determined. According to a previous study, it is speculated that the B2 phases should be partially ordered solid solutions^41^.

From the above results, it can be concluded that the Fe_*x*_(CrNiAl)_100−*x*_ alloys exhibit novel two-phase microstructures consisting of Ni-Al-rich B2-ordered nanoprecipitates embed in coherent Fe-Cr-rich BCC-disordered matrix. As the x increases, the structure of the B2 nanoprecipitates can transfer from a spinodal-like intertwined structure to a nanoparticle dispersed structure. Specifically, the Fe_55_(CrNiAl)_45_ and Fe_65_(CrNiAl)_35_ alloys contain a large density of highly dispersed nanoparticles in the matrix. These results provide an experimental evidence that non-equiatomic MEAs with one matrix element can form excellent microstructures.

## Discussion

The non-equiatomic MEA design we proposed breakthroughs the limitation of the existing HEA design, in which no matrix element is supposed to be used. As a result, a large number of new non-equiatomic MEAs can be obtained. For HEAs it is understood that the highest entropy will result in the lowest Gibbs free energy and thus, the highest phase stability. Then, what are the significances of MEA design? In our opinion, some MEAs with properly designed compositions may still have high phase stability, though they have reduced mixing entropies. For instance, the reported CrCoNi, FeNiCrCo and Fe_42_Mn_20_Ni_30_Co_6_Cr_2_ MEAs are all single-phase solid solutions, owing to their near-zero values of mixing enthalpy and small atomic size difference^37,42,43^. On the other hand, many MEAs may separate into multiple phases due to reduced phase stability resulting from reduced entropies, but they may benefit from the obtained multiple-phase structures. For example, compared with the most widely studied single-phase FeMnNiCoCr HEA, the dual-phase Fe_50_Mn_30_Co_10_Cr_10_ MEA can have better mechanical properties due to interface hardening and transformation hardening^40^. In comparison with the much-studied dual-phase FeNiCrCoAl HEA, the triple-phase Fe_33.3_Ni_33.3_Cr_16.7_Al_16.7_ MEA can exhibit better mechanical properties due to its composite effect of soft and hard phases^44^. It is believed that a number of MEAs with multiple-phase structures and excellent properties can be designed based on existing MEA and HEA systems. Moreover, for multicomponent alloys systems containing multiple phases, a proper non-equiatomic MEA design can be applied to optimize the size, morphology and distribution of phases for desired properties. The expensive elements can also be replaced by cost effective ones if possible in non-equiatomic MEA design, which will reduce the cost of alloys and facilitate their application. Therefore, in the future development of multicomponent alloys, the study of non-equiatomic MEAs is worth pursuing.

In order to interpret the phase separation behaviors in the Fe_*x*_(CrNiAl)_100−*x*_ alloys, we firstly analyze the interaction among the constituent elements in these alloys. Table 2 presents the physiochemical properties of the Fe, Cr, Ni and Al elements and the mixing enthalpies of binary systems containing these elements^29,45^. It can be seen that the mixing enthalpy of Ni-Al, Fe-Al and Cr-Al is −22 kJ/mol, −11 kJ/mol and −10 kJ/mol, respectively. Thus, they are all likely to form a B2 ordered phase^46,47^. However, the Ni and Al elements have the most negative mixing enthalpy as well as the largest difference of Pauling electronegativity. Therefore, they are most thermodynamically favored to form the Ni-Al-rich B2 phase. Since the Fe and Al elements also have large difference of Pauling electronegativity and large negative mixing enthalpy, the Fe element is thermodynamically favored to dissolve in the B2 phase. For the Cr element, although it also has quite large negative mixing enthalpy with Al, the difference of Pauling electronegativity between Cr and Al is the smallest. Hence, only a few concentration of Cr is dissolved in the B2 phase and most of Cr stays in the matrix, forming the BCC phase. Such separation behaviors of Fe-Cr-rich phase and Ni-Al-rich phase were also observed in the Fe-Cr-Co-Ni-Al HEA system^12,22^ and Fe-Cr-Co-Cu-Ni-Al HEA system^10,21^.

**Table 2:** Physiochemical properties of Fe, Cr, Ni and Al elements and mixing enthalpies of binary systems containing these elements calculated by Miedema’s model^45^.

Element		Fe	Cr	Ni	Al
Radius/Å		1.241	1.249	1.246	1.432
Pauling electronegativity		1.83	1.66	1.91	1.432
Valence electron concentration		8	6	10	3
*T*_m_ (K)		1808	2130	1726	933
Mixing enthalpies (kJ/mol)	Fe		−1	−2	−11
	Cr			−7	−10
	Ni				−22

The phase formation rules of HEAs have been widely studied through many parametric approaches, such as atomic size difference *δ*, mixing enthalpy Δ*H*_*mix*_, mixing entropy Δ*S*_*mix*_, valence electron concentration *VEC* and parameter *Ω*^25-29^. It has been demonstrated that, when *δ* ≤ 6.6% and *Ω* ≥ 1.1, the alloy can form simple solid solution phases^25^. Moreover, it was proposed that the stability of BCC and FCC phases can be predicted based on the values of *VEC*^28^. The FCC phase is stable if *VEC* ≥ 8, while the BCC phase is stable if *VEC* ≤ 6.87. When the value of *VEC* is between 6.87 and 8, a mixture microstructure containing both FCC and BCC phases is favored. For the Fe_*x*_(CrNiAl)_100−*x*_ alloys, the corresponding values of *δ*, Δ*H*_*mix*_, *T*_m_Δ*S*_*mix*_, *Ω* and *VEC* were calculated based on the expressions from reported studies^25,28^. As seen from Table 3, the values of *VEC* for the Fe_*x*_(CrNiAl)_100−*x*_ alloys are between 6.75 and 7.42. However, only BCC and B2 phases have been found in these alloys but no FCC phase exist. Thus, the formation of phases in the Fe_*x*_(CrNiAl)_100−*x*_ alloys can not be predicted by the existing *VEC* rules. In the studied alloys, the values of *δ* vary from 6.26% to 4.81% while the values of *Ω* are between 1.43 and 2.34. As predicted by the criteria of *Ω* > 1.1 and *δ* < 6.6% in HEAs, the alloys should form solid solution phases. However, these alloys form both BCC disordered phase and B2 ordered intermetallic phase. Hence, more exquisite criteria are needed to describe the separation of BCC and B2 phases.

**Table 3:** Parameters of Δ*H*_*mix*_, TΔ*S*_*mix*_, *Ω*, *δ*, and *VEC* calculated for the Fe_*x*_(CrNiAl)_100−*x*_ alloys.

Fe_*x*_(CrNiAl)_100−*x*_	Δ*H*_*mix*_(kJ/mol)	*T*_m_Δ*S*_*mix*_(kJ/mol)	*Ω*	*δ* × 100	*VEC*
*x* = 25	−13.25	19.00	1.43	6.26	6.75
*x* = 35	−11.57	18.91	1.63	6.01	6.92
*x* = 45	−9.86	18.18	1.84	5.70	7.08
*x* = 55	−8.13	16.84	2.07	5.30	7.25
*x* = 65	−6.37	14.88	2.34	4.81	7.42

The size, shape and distribution of Ni-Al-rich precipitates in the Fe_*x*_(CrNiAl)_100−*x*_ alloys have a close relationship with the concentration of Ni and Al elements. A high concentration of Ni and Al elements in the Fe25 alloy leads to the formation of a spinodal-like intertwined structure. At low concentrations of Ni and Al elements such as in Fe55 and Fe65 alloys, highly dispersed nanoparticle precipitates can be obtained. This provides a new approach to control phase separation in similar multicomponent alloys through simple variations in non-equiatomic compositions. The detailed formation processes of the Ni-Al-rich precipitates in the Fe_*x*_(CrNiAl)_100−*x*_ alloys are still unclear, which will be studied in the future.

In summary, here we propose a novel design of non-equiatomic medium-entropy alloys. Specifically, such alloys may contain one matrix element and several equiatomic alloying elements, while also having configurational entropies between 1 *R* and 1.5 *R*. Based on this new strategy, we successfully designed a series of Co-free Fe_*x*_(CrNiAl)_100−*x*_ medium-entropy alloys from the much-studied FeCrCoNiAl high-entropy alloy. The alloys exhibit novel two-phase microstructures consisting of Ni-Al-rich B2-ordered nanoprecipitates embed in Fe-Cr-rich BCC-disordered matrix. With the increase of *x*, the structure of the nanoprecipitates transfer from a spinodal-like intertwined structure to a nanoparticle dispersed structure. These phase separation behaviors can not be predicted by previous parametric approaches. Our findings provide a new route to design medium-entropy alloys and also demonstrate a possibility for designing nanostructured alloys in similar multicomponent alloy systems through simple variations in non-equiatomic compositions.

## Methods

In the processing of the Fe_*x*_(CrNiAl)_100−*x*_ alloys, the ingots were prepared by vacuum arc-melting under argon protection. The purity of raw Fe, Cr, Ni and Al metals is higher than 99.9 wt.%. All ingots were melted with electromagnetic stirring five times in order to homogenize their chemical compositions, and then the ingots were cooled naturally in the furnace. The crystal structures of the alloys were identified by an X-ray diffractometer (D/max-rb) with Cu radiation target. The scan range 2*θ* is from 20° to 100° using a scanning step size of 0.026°. The microstructures of the alloys were characterized by a scanning electron microscopy (Quanta 200FEG). The chemical compositions of phases in the alloys were analyzed by a transmission electron microscopy (Talos F200x) using an high-angle annular dark-field detector and an energy dispersive spectrometry (EDS).

## References

[1] Yeh JW (2004). Nanostructured high-entropy alloys with multiple principal elements: Novel alloy design concepts and outcomes. Adv. Eng. Mater..

[2] Cantor B (2004). Microstructural development in equiatomic multicomponent alloys. Mater. Sci. Eng. A.

[3] Yeh JW (2013). Alloy design strategies and future trends in high-entropy alloys. JOM..

[4] Gao, M. C., Yeh, J. W., Liaw, P. K. & Zhang, Y. High-entropy alloys: fundamentals and applications. Springer (2016).

[5] Miracle DB, Senkov ON (2017). A critical review of high entropy alloys and related concepts. Acta Mater..

[6] Ye YF (2016). High-entropy alloy: challenges and prospects. Mater. Today.

[7] Laurent-Brocq M (2015). Insights into the phase diagram of the CrMnFeCoNi high entropy alloy. Acta Mater..

[8] Wang Q (2016). A cuboidal B2 nanoprecipitation-enhanced body-centered-cubic alloy Al_0.7_CoCrFe_2_Ni with prominent tensile properties. Scripta Mater..

[9] Shun TT, Hung CH, Lee CF (2010). Formation of ordered/disordered nanoparticles in FCC high entropy alloys. J. Alloy. Comp..

[10] Tung CC (2007). On the elemental effect of AlCoCrCuFeNi high-entropy alloy system. Mater. Lett..

[11] Senkov ON (2011). Mechanical properties of Nb_25_Mo_25_Ta_25_W_25_ and V_20_Nb_20_Mo_20_Ta_20_W_20_ refractory high entropy alloys. Intermetallics.

[12] Kao YF (2009). Microstructure and mechanical property of as-cast,-homogenized, and-deformed Al_x_CoCrFeNi (0 ≤ x ≤ 2) high-entropy alloys. J. Alloy. Comp..

[13] Zhou YJ (2007). Solid solution alloys of AlCoCrFeNiTi_x_ with excellent room-temperature mechanical properties. Appl. Phys. Lett..

[14] Hsu CY (2011). On the superior hot hardness and softening resistance of AlCoCr_x_FeMo_0.5_Ni high-entropy alloys. Mater. Sci. Eng. A.

[15] Chuang MH (2011). Microstructure and wear behavior of Al_x_Co_1.5_CrFeNi_1.5_Ti_y_ high-entropy alloys. Acta Mater..

[16] Lin CM, Tsai HL (2011). Evolution of microstructure, hardness, and corrosion properties of high-entropy Al_0.5_CoCrFeNi alloy. Intermetallics.

[17] Zhang Y (2013). High-entropy alloys with high saturation magnetization, electrical resistivity, and malleability. Sci. Rep..

[18] Zhang Y (2014). Microstructures and properties of high-entropy alloys. Prog. Mater. Sci..

[19] Gludovatz B (2014). A fracture-resistant high-entropy alloy for cryogenic applications. Science.

[20] Jiang L (2015). Effect of Mo and Ni elements on microstructure evolution and mechanical properties of the CoFeNi_x_VMo_y_ high entropy alloys. J. Alloy. Comp..

[21] Singh S (2011). Decomposition in multi-component AlCoCrCuFeNi high-entropy alloy. Acta Mater..

[22] Manzoni A (2013). Phase separation in equiatomic AlCoCrFeNi high-entropy alloy. Ultramicroscopy.

[23] Cheng CY, Yeh JW (2016). High thermal stability of the amorphous structure of Ge_x_NbTaTiZr(x = 0.5,1) high-entropy alloys. Mater. Lett..

[24] Chattopadhyay C (2016). Critical evaluation of glass forming ability criteria. Mater. Sci. Tech..

[25] Yang X, Zhang Y (2012). Prediction of high-entropy stabilized solid-solution in multi-component alloys. Mater. Chem. Phy..

[26] Guo S (2013). More than entropy in high-entropy alloys: forming solid solutions or amorphous phase. Intermetallics.

[27] Otto F (2013). Relative effects of enthalpy and entropy on the phase stability of equiatomic high-entropy alloys. Acta Mater..

[28] Guo S (2011). Effect of valence electron concentration on stability of fcc or bcc phase in high entropy alloys. Appl. Phys..

[29] Guo S, Liu CT (2011). Phase stability in high entropy alloys: formation of solid-solution phase or amorphous phase. Prog. Nat. Sci.: Mater. Inter..

[30] Ranganathan S (2003). Alloyed pleasures: multimetallic cocktails. Current science.

[31] Nmrl A, Igcar B (2015). High entropy alloys: a renaissance in physical metallurgy. Current Science.

[32] Murty, B. S., Yeh, J. W., Ranganathan S. High-entropy alloys. Butterworth-Heinemann (2014).

[33] Pradeep KG (2015). Non-equiatomic high entropy alloys: Approach towards rapid alloy screening and property-oriented design. Mater. Sci. Eng. A.

[34] Raabe D (2015). From High-Entropy Alloys to High-Entropy Steels. . steel research int..

[35] Laurent-Brocq M (2016). From high entropy alloys to diluted multi-component alloys: Range of existence of a solid-solution. Mater. Des..

[36] Li, B. S. Microstructure formation mechanisms and properties of micro/nano structured high-entropy alloys and composites. Ph. D. thesis. School of Materials Science and Engineering, HIT, China, 2012.

[37] Ma D (2015). Phase stability of non-equiatomic CoCrFeMnNi high entropy alloys. Acta Mater..

[38] Yao MJ (2014). A novel, single phase, non-equiatomic FeMnNiCoCr high-entropy alloy with exceptional phase stability and tensile ductility. Scripta Mater..

[39] Stepanov ND (2015). Tensile properties of the Cr-Fe-Ni-Mn non-equiatomic multicomponent alloys with different Cr content. Mater. Des..

[40] Li Z (2016). Metastable high-entropy dual-phase alloys overcome the strength-ductility trade-off. Nature.

[41] Santodonato LJ (2015). Deviation from high-entropy configurations in the atomic distributions of a multi-principal-element alloy. Nature comm..

[42] Gludovatz B (2016). Exceptional damage-tolerance of a medium-entropy alloy CrCoNi at cryogenic temperatures. Nature comm..

[43] Wu Z (2014). Recovery, recrystallization, grain growth and phase stability of a family of FCC-structured multi-component equiatomic solid solution alloys. Intermetallics.

[44] Dong Y (2016). A multi-component AlCrFe_2_Ni_2_ alloy with excellent mechanical properties. Mater. Lett..

[45] Takeuchi A, Inoue A (2005). Classification of bulk metallic glasses by atomic size difference, heat of mixing and period of constituent elements and its application to characterization of the main alloying element. Mater. Trans..

[46] Helander T, Tolochko O (1999). An experimental investigation of possible B2-ordering in the Al-Cr system. J. Phase Equilibria.

[47] Hu R, Nash P (2006). Experimental enthalpies of formation of compounds in Al-Ni-X systems. J. Mater. Sci..

